# An Information-Theoretic Account of Semantic Interference in Word Production

**DOI:** 10.3389/fpsyg.2021.672408

**Published:** 2021-05-31

**Authors:** Richard Futrell

**Affiliations:** Department of Language Science, University of California, Irvine, Irvine, CA, United States

**Keywords:** language production, information theory, bounded rationality, semantic interference effect, Stroop, rate-distortion

## Abstract

I present a computational-level model of semantic interference effects in online word production within a rate–distortion framework. I consider a bounded-rational agent trying to produce words. The agent's action policy is determined by maximizing accuracy in production subject to computational constraints. These computational constraints are formalized using mutual information. I show that semantic similarity-based interference among words falls out naturally from this setup, and I present a series of simulations showing that the model captures some of the key empirical patterns observed in Stroop and Picture–Word Interference paradigms, including comparisons to human data from previous experiments.

## 1. Introduction

In cognitive science and related fields, **bounded rationality** is the idea that our cognitive systems are designed to take actions that are approximately optimal, given that only limited computational resources are available for calculating the optimal action (Simon, 1955, 1972; Kahneman, 2003; Howes et al., 2009; Lewis et al., 2014; Gershman et al., 2015; Lieder and Griffiths, 2019). The idea is appealing because it maintains the mathematical precision of theories based on rationality, while avoiding the paradoxes and empirical shortcomings that come from claiming that human beings act in ways that are entirely rational. There has been recent interest in formalizing bounded rationality within the mathematical framework of rate–distortion theory (Berger, 1971; Cover and Thomas, 2006) with applications to cognitive science (Sims, 2016, 2018; Zaslavsky et al., 2018; Gershman, 2020).

In this paper, I apply rate–distortion theory to derive a model of online word production. The goal is to model the difficulty of online word production, as measured using psychometric dependent variables, such as reaction time and rates and patterns of errors. The main contribution of this paper is to show that rate–distortion theory generically predicts the well-documented **semantic interference effects** that a subject experiences when trying to produce a target word in the presence of a semantically related distractor. For example, the Stroop task famously exhibits interference (Stroop, 1935): given a stimulus, such as the word **BLUE** printed in red ink, and an instruction to name the color of the ink, it is hard to produce “red” because of interference from the similar word “blue.” A similar kind of interference is present in the Picture–Word Interference task, where a drawing must be named in the presence of a superimposed distractor word (Lupker, 1979; Starreveld and La Heij, 2017). Beyond the basic interference effect, I show that rate–distortion theory predicts a number of key phenomena observed in such tasks.

## 2. Background: Rate–Distortion Theory of Control

### 2.1. Bounded Rationality

Ultimately, our cognitive systems implement an **action policy**: a function from sensory inputs to motor outputs. For example, an animal might see another animal and decide among a large set of possible actions, including attacking, approaching, ambushing, fleeing, etc. In general, we can conceive of an action policy as a stochastic function mapping states *S* (including perceptual, physiological, and memory information) to motor actions *A*:

$$\begin{array}{l} q:S\to A. \end{array}$$

We can also think of the policy as a *probability distribution* on actions given states, where *q*(*a*|*s*) denotes the probability of taking action *a* in state *s*.

A **bounded-rational action policy** is a policy that chooses an action to maximize some measure of reward, or equivalently, to minimize the cost of the *consequences* of taking a certain action in the world, subject to a constraint on the computational resources used in finding and implementing this action. These resources include factors, such as time—in many circumstances, it may be more important to act quickly than to take the time to compute the best action—as well as physiological resources, such as the energy required to perform computations. Formally, letting *D*(*s, a*) represent the **action cost** or the cost of the consequences of taking action *a* in state *s*, and letting *C*(*s, a*) denote the **computation cost** required to compute the action *a* given state *s*, then the overall cost for a policy *q* can be written as

(1) $$\begin{array}{l} L\left(q\right)=\langle D\left(s,a\right)+\frac{1}{\gamma }C\left(s,a\right)\rangle , \end{array}$$

where 〈·〉 denotes an average over the joint probability distribution on states and actions given those states *p*(*s*)*q*(*a*|*s*), and $\frac{1}{\gamma }$ is a scalar value which indicates how much a unit of computation cost *C* should be weighed against a unit of action cost *D*. The scalar γ can also be viewed as a parameter giving the amount of resources available for computation: high γ means that the agent is willing to perform a lot of computation in order to minimize the action cost *D*.

The expression L(q) in (1) is called the **control objective**, and a bounded optimal action policy is derived by minimizing it:

$$\begin{array}{l} q_{\text{bounded rational}}=\arg\underset{q}{\min}L\left(q\right), \end{array}$$

where the minimization is over the set of all possible policies. The bounded-rational policy reduces to the fully rational policy in the case when computation costs have negligible importance, i.e., $\frac{1}{\gamma }\to 0$ in Equation (1).

Without further specifications, the theory of bounded rationality goes no farther than the formalization above. Given a set of cost functions, the bounded rational action policy is derived as the solution to a multi-objective minimization problem involving those cost functions. The theory only makes precise predictions when the cost functions and their relative weights are further specified. Below, we will see how we can do this in a principled way using tools from information theory.

### 2.2. Rate–Distortion Theory

Rate-distortion theory is the mathematical theory of lossy communication and compression, a subfield of information theory. It provides mathematical tools to answer questions like: if I want to transmit a picture of a zebra to you, and I do not have the capacity to send it to you perfectly, how can I encode the image such that your received picture looks approximately like what I sent? This problem involves two constraints: (1) my capacity to transmit information (called **rate**), and (2) a measure of how much your received picture differs from my picture (this measure is called **distortion**). Rate–distortion theory describes the problem of finding a data encoding which minimizes the distortion subject to a constraint on the rate.

The link between rate–distortion theory and bounded rational action policies was not immediately clear, although the original paper on rate–distortion theory did note a connection with control theory (Shannon, 1959, p. 350). The key insight that has enabled researchers to link these two theories is that rate–distortion theory can be applied to constrain the perception–action loop. The idea is to treat an action policy as a communication channel from sensory input to motor output. Then the action cost *D* in Equation (1) is the distortion, and the computation cost *C* in Equation (1) is the rate. This connection was introduced first in the economics literature by Sims (2003, 2005, 2010) under the name **rational inattention**: the idea being that an agent might decide not to attend to certain information because the computational resources required to sustain that attention are not worth the investment. The idea was then picked up in the robotics, cybernetics, machine learning, and psychology literature (van Dijk et al., 2009; Tishby and Polani, 2011; Rubin et al., 2012; Ortega and Braun, 2013; Genewein et al., 2015; Sims, 2016, 2018; Gershman and Bhui, 2020, among others).

In the **rate–distortion theory of control** (RDC), a bounded-rational action policy is derived by minimizing the following control objective:

(2) $$\begin{array}{l} L\left(q\right)=\langle D\left(s,a\right)\rangle +\frac{1}{\gamma }I\left[S:A\right], \end{array}$$

where *D*(*s, a*) is the distortion or action cost for taking action *a* in state *s*, and *I*[*S* : *A*] denotes the **mutual information** between the random variables *S* representing the state and *A* representing the action policy:

$$\begin{array}{l} I\left[S:A\right]=\langle \log\frac{q\left(a|s\right)}{q\left(a\right)}\rangle , \end{array}$$

where the probability *q*(*a*) is the marginal probability of taking action *a* under the policy *q*, averaging over all states:

$$\begin{array}{l} q\left(a\right)=\underset{s}{\sum }p\left(s\right)q\left(a|s\right). \end{array}$$

The substantive claim of the RDC is that computation costs should be modeled as the mutual information between states and actions *I*[*S* : *A*]. This quantity can be interpreted as the amount of information that must be extracted from *S* in order to specify *A* (Sims, 2003), or as the information throughput of a controller implementing the policy *q*(*a*|*s*) (Fan, 2014). I will argue below that this is a natural measure of computation cost, and that it subsumes many other measures.

I summarize four converging motivations for the use of the mutual information between states and actions *I*[*S* : *A*] (and related measures, such as relative entropy) as a measure of computation cost. I provide pointers into the literature for the full forms of these arguments. See also Zénon et al. (2019, section 4) for a comprehensive discussion and review.

1. **Computation time**. The mutual information reflects the *search time* taken to find the action *A* given state *S* by a rejection sampling algorithm. When the mutual information *I*[*S* : *A*] is lower, the correct action can be found using fewer samples from *q*(*a*) (Braun and Ortega, 2014, section 2).
2. **Algorithmic complexity**. The mutual information reflects how many bits of information an agent must store to remember the policy, or how many bits of information an agent needs to observe to learn the policy. This argument is presented in a PAC-Bayes framework by Rubin et al. (2012), who also show that action policies with a mutual information penalty are less prone to overfitting to their immediate environment.
3. **Free energy**. The RDC objective in Equation (1) is technically a **free energy** functional (Ortega and Braun, 2013), bringing the theory in line with neuroscientific theories of brain function formulated in terms of minimizing free energy (Friston, 2010).
4. **Congruence with empirically-observed laws of behavior**. Information-theoretic models of cognitive control have proposed that the time taken to initiate an action should be proportional to the amount of information required to specify that action (Fan, 2014). We can derive well-validated empirical laws of behavior under this assumption. For example, Hick's Law is the observation that the time taken to decide among a set of actions *A* is directly proportional to the logarithm of the number of possible actions log|*A*| (Hick, 1952; Hyman, 1953). The RDC computation cost *I*[*S* : *A*] reduces to log|*A*|, yielding Hick's Law, in the case where (1) an agent is deciding among a set of actions *A*, (2) the default policy *q*(*a*) is uninformative about which action to take, and (3) the state-dependent policy *q*(*a*|*s*) specifies the desired action deterministically.

In summary, there is a convergence among a number of previous intuitive notions of computation cost, all of which point toward *I*[*S* : *A*] as a reasonable measure. In addition to these theoretical arguments, a growing neuroscience literature has linked information measures, such as *I*[*S* : *A*] to brain activity in the prefrontal cortex (Koechlin and Summerfield, 2007; Fan, 2014).

The form of the RDC objective in Equation (2) is only the simplest member of a family of possible control objectives. In reality, a cognitive agent must integrate information from many different inputs and produce motor output on many different actuators. Each input and each motor output can be associated with its own channel, with its own information-based penalty. Multiple input channels can be modeled by adding further weighted mutual information terms to Equation (2) (for example, see van Dijk and Polani, 2011, 2013; Genewein et al., 2015). In fact, we will see that our model of Picture–Word Interference requires at least two input channels: a top-down goal signal and a bottom-up perceptual signal.

### 2.3. Solutions to the RDC Objective

The policies admitted under the rate–distortion theory of control have a common mathematical form. The minima of Equation (2) obey the following equations:

(3) $$\begin{array}{l} q\left(a|s\right)=\frac{1}{Z\left(s\right)}q\left(a\right)\exp\left\{-\gamma D\left(s,a\right)\right\}\\ \;q\left(a\right)=\underset{s}{\sum }p\left(s\right)q\left(a|s\right)\\ \;Z\left(s\right)=\underset{a}{\sum }q\left(a\right)\exp\left\{-\gamma D\left(s,a\right)\right\}. \end{array}$$

Note that the Equation (3) do not specify a policy uniquely. The equations are called self-consistent, meaning that any *q*(*a*|*s*), *q*(*a*), and *Z*(*s*) jointly constitute a minimum of the control objective as long as they satisfy the three equations simultaneously. In general, multiple solutions can exist. A numerical solution to the equations can be found by starting with a random value of *q*(*a*|*s*), then evaluating the equations iteratively until a fixed point is reached.

One generalization that we can deduce immediately from this system of equations is that RDC policies favor re-use of common actions. We can see this because the factor *q*(*a*) in Equation (3) will be high for actions that are taken frequently across all states. Therefore, these actions will be preferred, sometimes in lieu of the action that would be more appropriate in a particular state *s*. Intuitively, the factor *q*(*a*) represents a “habit”: a propensity to take a certain action regardless of the present context (van Dijk and Polani, 2013; Wood and Rünger, 2016; Gershman, 2020).

### 2.4. Link to Behavioral Measures

The RDC describes the derivation of bounded-rational action policies, but does not immediately make predictions about the timing of these actions nor other behavioral and neural dependent measures that are commonly deployed in the study of cognitive control and language production. A linking hypothesis is required from the mathematical policy *q*(*a*|*s*) to predictions about dependent measures, such as reaction time, the usual measure of difficulty in word production studies.

There are a number of perspectives in the psychological literature on the relationship between reaction times (RTs) and information-theoretic measures of complexity (Laming, 1968, 2003; Luce, 2003; Ortega and Braun, 2013; Fan, 2014; Zénon et al., 2019; Lynn et al., 2020). The simplest possible hypothesis is that the time required to initiate an action is linearly proportional to the amount of computation that needs to be done to select the action. For example, Fan (2014) conceptualizes cognitive control as the means by which uncertainty about the output action is reduced at a constant rate in terms of bits per millisecond. I adopt this linking hypothesis here, with a modification to account for the fact that the computation required to select an action breaks into multiple parts, which I call computation cost and decision cost:

1. **Computation cost**. The computation required to produce the action policy *q*(*a*|*s*). This is equal to the cost term in the control objective L that generates *q*(*a*|*s*). For example, given the control objective in Equation (2), the average computation cost is the mutual information $I\left[S:A\right]=\langle \log\frac{q\left(a|s\right)}{q\left(a\right)}\rangle$. For a particular action *a* in state *s*, the cost is the pointwise mutual information $\log\frac{q\left(a|s\right)}{q\left(a\right)}$. This notion of computation cost combines Zénon et al. (2019)'s notions of “perceptual cost” and “automatic cost.” For human behavioral work relating this notion of computation cost to computation time, see Ortega and Stocker (2016) and Schach et al. (2018).
2. **Decision cost**. A policy *q*(*a*|*s*) is a probability distribution on actions, but in any given state, an agent must take a single action. Decision cost is the cost associated with selecting a single action a^*^ from a distribution *q*(*a*|*s*); it represents a decision that still needs to be made (perhaps randomly) after considering state information. I take decision cost to be equal to the KL divergence from *q*(*a*|*s*) to a delta distribution specifying a single action a^*^:
  $$\begin{array}{l} D_{\text{KL}}\left[\delta_{aa^{*}}\|q\left(a|s\right)\right]=\langle \log\frac{\delta_{aa^{*}}}{q\left(a|s\right)}\rangle \\ \;=-\log q\left(a^{*}|s\right), \end{array}$$
  where $\delta_{aa^{*}}$ is a Kronecker delta function (equal to 1 when *a* = a^*^ and 0 otherwise). Thus, decision cost comes out to be the surprisal (negative log probability) of the action a^*^ given the state *s* under the action policy.

It stands to reason that both computation cost and decision cost make contributions to dependent measures, such as reaction time, although perhaps not according to a simple function. In this work I will present computation and decision cost in terms of bits of information, and where appropriate I will discuss their possible translation into observable dependent measures.

There have been other, more complex proposals about the link between RDC policies and observable measures, such as reaction time. For example, Ortega and Braun (2013, p. 10–11) link RDC policies to drift–diffusion models of choice behavior (Bogacz et al., 2006). While I do not pursue these other linking hypotheses here, they could provide different perspectives or more precise predictions in future work.

### 2.5. Level of Analysis

RDC as applied to word production is a computational-level theory in Marr's sense (Marr, 1982), meaning that it attempts to model the problem that is being solved in language production. Because it is stated at this level of abstraction, it is not necessarily in conflict with existing more mechanistic models of word production. RDC states simply that the cognitive cost of taking certain actions is determined by a trade-off of minimizing action cost while also minimizing information-processing costs, measured using mutual information. This trade-off might be implemented in terms of spreading activation in networks with constrained topology, production rules, etc. Nevertheless, it will be interesting to see where the predictions of more mechanistic theories diverge from those of the more abstract RDC.

To sum up this section, I have presented the rate–distortion theory of control (RDC) as a model of bounded-rational action. Below, I will present a new application of this model to model human word production, which exhibits a property of the model which has not previously been explored. In particular, I will show that similarity-based interference effects, which are common in word production as well as other aspects of cognition, arise as a generic prediction of RDC models.

## 3. Interference in the Rate–Distortion Theory of Control

In this section I will demonstrate the basic mechanism by which RDC predicts similarity-based interference effects.

### 3.1. The Empirical Phenomena

The term **similarity-based interference** encompasses a large number of phenomena in human perception, action, and memory. It refers to the idea that percepts, actions, or memories are confused for each other when they are “similar” according to some metric (Shepard, 1987), that is, when they share features or associated cues. Furthermore, there may be increased latency in identifying a percept, retrieving information from memory (Jäger et al., 2017), or initiating in action (Stroop, 1935) in the presence of some “similar” distractor. Capturing similarity-based interference is a key goal of cognitive models, including those based on cue-based retrieval, spreading activation, and production rules (Watkins and Watkins, 1975; Ratcliff, 1978; Anderson and Lebiere, 1998; Roelofs, 2003).

### 3.2. RDC Account

Similarity-based interference arises generically in RDC models because the action cost *D*(*s, a*) naturally defines a similarity metric among actions, an insight used by Sims (2018) in his model of generalization in absolute identification tasks. The function *D*(*s, a*) gives the cost of taking action *a* in state *s*. Two actions are similar when they have similar cost, that is, when there is low cost for failing to distinguish them. Accordingly, we can define a distance metric between two actions. In state *s*, let *a*_*s*_ be the action with minimal cost, and *a*_*d*_ be any other action. The state-dependent distance metric among actions can be defined as a function

$$\begin{array}{l} d\left(a_{s},a_{d}\right)=D\left(s,a_{d}\right)-D\left(s,a_{s}\right). \end{array}$$

This distance metric^1^ will play the role of the distortion metric in rate–distortion theory.

Now that we have a distance metric among actions, we can see that interference effects arise even in the simplest formulation of the RDC. Suppose the control system is attempting to solve the following problem: in a state *s* (for example, seeing a picture of an apple), there is a single unique target action *a*_*s*_ corresponding to that state (for example, saying the word “apple”). The agent is attempting to generate the right target action in state *s*. In this setting, RDC predicts generally that the probability that any two actions (e.g., words) *a*_*s*_ and *a*_*d*_ are confused will increase as they get closer in the distance metric *d*(*a*_*s*_, *a*_*d*_)—thus predicting similarity-based interference among competitors.

More formally, let the control objective be

(4) $$\begin{array}{l} L\left(q\right)=\langle d\left(a_{s},a\right)\rangle +\frac{1}{\gamma }I\left[S:A\right]. \end{array}$$

This equation expresses that the agent will try to minimize the average distance between the selected action *a* and the target action *a*_*s*_, subject to a computation cost of $\frac{1}{\gamma }$ units per bit of information from the states *S* used to specify actions *A*. Then following the logic in Equation (3), the bounded-rational policy has the form

(5) $$\begin{array}{l} q\left(a|s\right)=\frac{1}{Z\left(s\right)}q\left(a\right)\exp\left\{-\gamma d\left(a_{s},a\right)\right\}\\ \;q\left(a\right)=\underset{s}{\sum }p\left(s\right)q\left(a|s\right)\\ \;Z\left(s\right)=\underset{a}{\sum }q\left(a\right)\exp\left\{-\gamma d\left(a_{s},a\right)\right\}. \end{array}$$

This policy exhibits exponentially-decaying interference effects as a function of the distance *d*(*a*_*s*_, *a*). To see this, let's simplify the setting, considering a scenario where there are only two possible actions given a state *s*: the target action *a*_*s*_ and a single distractor *a*_*d*_. Plugging in to Equation (5), we find that the probability of the target action *a*_*s*_ in state *s* is given by a logistic curve^2^:

(6) $$\begin{array}{l} q\left(a_{s}|s\right)=\frac{1}{1+\frac{q\left(a_{d}\right)}{q\left(a_{s}\right)}\exp\left\{-\gamma d\left(a_{s},a_{d}\right)\right\}}. \end{array}$$

**Figure 1:**
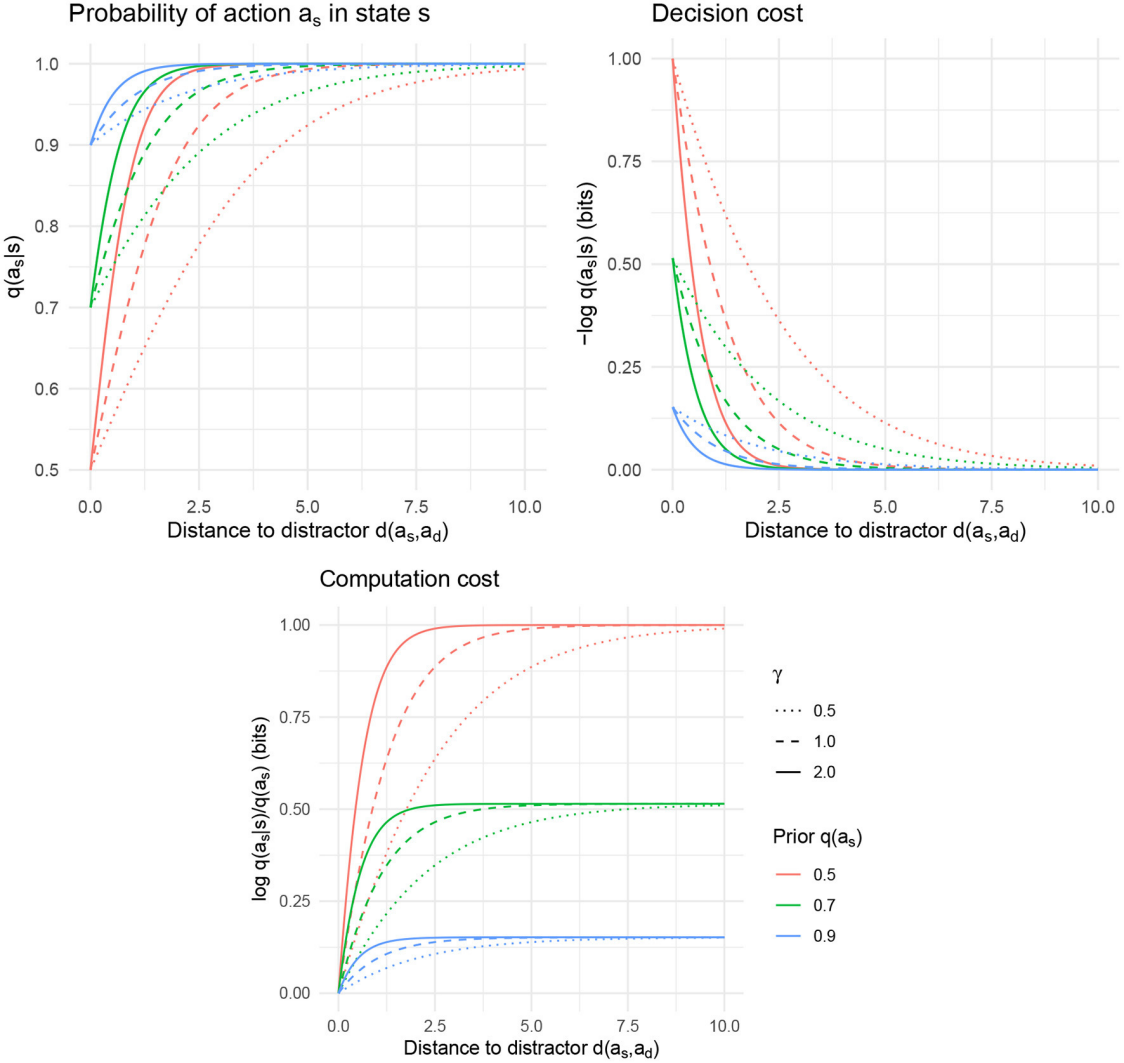
Interference between a target action *a*_*s*_ and a distractor *a*_*d*_ as a function of the distance *d*(*a*_*s*_, *a*_*d*_), for varying values of resource parameter γ and the a priori probability *q*(*a*_*s*_). **(Top left)** The probability *q*(*a*_*s*_|*s*) of taking the appropriate action *a*_*s*_ in state *s*. **(Top right)** The decision cost − log *q*(*a*_*s*_|*s*), which is high when *a*_*s*_ and *a*_*d*_ have low semantic distance. **(Bottom)** The computation cost $\log\frac{q\left(a_{s}|s\right)}{q\left(a_{s}\right)}$.

The curve is illustrated in Figure 1. The important part of Equation (6) is the second term in the denominator, which represents the effect of interference between the target action *a*_*s*_ and the distractor action *a*_*d*_. As this interference term gets larger, the probability of the target action *q*(*a*_*s*_|*s*) gets smaller. This interference term is large when (1) the distractor action *a*_*d*_ is a priori likely, and (2) the distractor action *a*_*d*_ is close to the target action *a*_*s*_.

An agent with a control objective as in Equation (4) will therefore show similarity-based interference in terms of errors in the action taken. This interference also manifests in decision cost for action *a*_*s*_:

$$\begin{array}{l} \text{Decision cost}=-\log q\left(a_{s}|s\right)\\ \;=\log\left(1+\frac{q\left(a_{d}\right)}{q\left(a_{s}\right)}\exp\left\{-\gamma d\left(a_{s},a_{d}\right)\right\}\right), \end{array}$$

visualized in Figure 1. This function decreases as *d*(*a*_*s*_, *a*_*d*_) increases. The computation cost, on the other hand, decreases when *d*(*a*_*s*_, *a*_*d*_) decreases, reflecting the main mechanism by which similarity-based interference arises in this model: at small distances *d*(*a*_*s*_, *a*_*d*_), the policy achieves lower computation cost at the expense of decreased accuracy in the action selected.

Applying this logic to word production, we predict interference effects among semantically similar production targets when both are likely actions given the agent's state. Consider a state where a person sees a picture of an apple, and the words “apple” and “pear” are both a priori likely for some reason. This corresponds to target action *a*_*s*_ = say “apple” and distractor action *a*_*d*_ = say “pear”, with *q*(*a*_*s*_) and *q*(*a*_*d*_) both high, and *d*(*a*_*s*_, *a*_*d*_) low. A bounded-rational agent will erroneously say “pear” in this state more often than if the distractor were something less similar, such as $a_{d}^{\prime }=\text{say “car”}$; furthermore, the action *a*_*s*_ = say “apple” can only be produced at higher decision cost due to the presence of the distractor. The reason is that when the distractor is “car,” the relevant distance is $d\left(a_{s},a_{d}^{\prime }\right)\gg d\left(a_{s},a_{d}\right)$, leading to a lower probability of confusion in the action policy.

This example embodies the core logic of the RDC account of interference. Below, I will demonstrate this logic in a more thoroughly worked out model of the Stroop/Picture–Word Interference Task including fits to human behavioral data. That simulation will require a more involved control model, but the underlying cause of similarity-based interference remains the same as in this example.

## 4. Model of Picture–Word Interference

Here, I show that RDC can capture some of the major characteristics of semantic interference in the Picture–Word Interference task.

### 4.1. Phenomena

**Figure 2:**
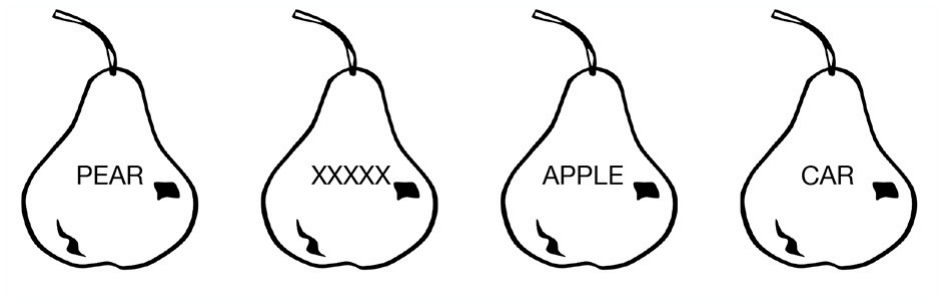
Conditions of a Picture–Word Interference experiment. From left to right: the **congruent**, **neutral**, **semantic**, and **unrelated** conditions (see text).

**Picture–Word Interference** (PWI) is one of the most well-studied phenomena in language production and cognitive control (Schriefers et al., 1990; Damian and Martin, 1999; Bürki et al., 2020). The task evokes similarity-based interference in picture naming by superimposing a text word over an image, and asking a subject to name the image (Lupker, 1979). Examples are shown in Figure 2. The **Stroop task** is closely related (Stroop, 1935; MacLeod, 1991; van Maanen et al., 2009; Starreveld and La Heij, 2017): in this task, a word, such as **BLUE** is presented in red ink, and subjects are asked to name the color of the ink.

The hallmark PWI effect is that subjects are slower to name the image in the presence of a superimposed word which is semantically categorically related to the image (the **semantic** condition in Figure 2), as compared to their reaction times when the superimposed text is a neutral string, such as XXXXX (the **neutral** condition in Figure 2). Furthermore, reaction times are fastest when the superimposed word is the same as the name of the image (the **congruent** condition), and if the superimposed text is a semantically unrelated word (the **unrelated** condition), reaction times are somewhere between the neutral and semantic conditions. “Semantic interference” in the PWI task refers to this additional slowdown and increased probability of error for the semantic condition relative to the unrelated condition.

Many PWI and Stroop experiments include only a neutral or an unrelated condition, rather than all four of these conditions, which has resulted in some variance in terms of the size of the reported interference effect (MacLeod, 1991). The neutral and unrelated conditions are referred to together as the **baseline** conditions, and the semantic and unrelated conditions are referred to together as the **incongruent** conditions.

### 4.2. Related Work

Because of its empirical robustness and (apparent) conceptual simplicity, PWI and Stroop tasks have been the target of many computational cognitive models throughout the past three decades, and subject to intense controversies about the mechanism that gives rise to the observed interference effect.

The main controversy in the literature is over whether PWI effects are driven by a competitive process during lexical selection, where multiple responses are competing for priority, resulting in slowdown (Roelofs, 1992; Levelt et al., 1999; Damian and Bowers, 2003; Belke et al., 2005; Abdel Rahman and Melinger, 2009) or by the need to exclude the distractor from an articulatory buffer (for example, Mahon et al., 2007). The most extensively documented and tested model of PWI is WEAVER++ (Roelofs, 1992, 2003; Levelt et al., 1999), a model of word production based on production rules and spreading activation where similarity-based interference emerges due to competition in lexical selection.

In contrast to existing computational models, the RDC account of interference in word production is a computational-level model which works by specifying only the problem that is being solved by the cognitive system, without making any commitments to algorithmic-level details (Marr, 1982). The theory and its assumptions are specified completely by (1) the control objective, which is the mathematical statement of the problem that the cognitive system is trying to solve, and (2) the linking function from cognitive costs to observables, such as RT.

As we will see, the control objective that reproduces PWI effects specifies only that there is some computational bottleneck involved in integrating information from bottom-up sensory input and top-down behavioral goals—whether this bottleneck happens in lexical selection, articulation, etc. is unspecified. The computational bottleneck might arise more mechanistically due to dynamics of spreading activation, competing production rules, etc. The question of whether the interference effect arises because of competition or response exclusion does not arise at this level of abstraction.

I am aware of two previous information-theoretic models of the Stroop task. Zénon et al. (2019) present a model of information-processing costs in the Stroop task which predicts that performing an unusual goal (i.e., naming a picture rather than reading a word) results in increased difficulty. Their model does not use bounded-optimal policies and does not account for semantic interference. Also, Christie (2019) models the RT response distribution for congruent, semantic, and neutral trials in a Stroop task using an information-theoretic model in which conflicting control signals are superposed and must be decoded at high cost. This model involves a policy which receives noisy bottom-up and top-down signals and must decide on an action. While this model is based on a noisy channel, rather than rate–distortion theory, it is fundamentally similar to the model presented here because it involves rational action under cognitive constraints modeled using information theory.

### 4.3. RDC Account

A full model of PWI requires a more complex setup than the simple interference example above. In particular, whereas the interference model given by Equation (4) involved a policy conditional only on an input state, a full model of PWI requires a policy conditional on *two* inputs: a perceptual state and a top-down behavioral goal.

**Figure 3:**
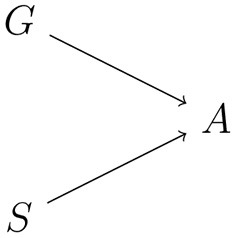
Schematic of an action policy where the behavioral goal *G* and the perceptual state *S* jointly determine the output action *A*.

To model PWI, let *G* be a random variable representing a speaker's top-down goals, i.e., whether the goal is to name a picture/color or to read a word. That is, *G* is a random variable taking values in the set {name, read}. Let *S* be a random variable representing a speaker's perceptual state—that is, the particular word and picture that the speaker is looking at. A speaker then implements a bounded-rational production policy on actions given goals and perceptual states *q*(*a*|*g, s*), subject to information-processing costs. The structure of the model is shown in Figure 3.

As the output action is jointly determined by the behavioral goal *G* and the perceptual state *S*, the total mutual information between the inputs to the policy and the output action is given by the formula

(7) $$\begin{array}{l} I\left[G,S:A\right]=\langle \log\frac{q\left(a|g,s\right)}{q\left(a\right)}\rangle . \end{array}$$

This quantity gives the total amount of information in the behavioral goal *G* and perceptual state *S* that the policy uses in order to specify the action *A*. The simplest RDC policy would simply take Equation (7) as the computation cost. However, it turns out that in order to model the PWI task, we need to assign different levels of cost to information coming from the two sources, *G* and *S*.

In order to do so, we must first break the quantity in Equation (7) down into two parts, reflecting the contributions of *S* and *G*. Using the chain rule for mutual information (Cover and Thomas, 2006, p. 24, Theorem 2.5.2), we can write:

$$\begin{array}{r} \underset{\text{information transmitted from}\;G\;\text{and}\;S\;\text{to specify}\;A}{\underset{︸}{I\left[G,S:A\right]}}=\underset{\text{information from}\;S}{\underset{︸}{I\left[S:A\right]}}\\ +\underset{\text{information from}\;G\;\text{conditional on}\;S}{\underset{︸}{I\left[G:A|S\right]}}, \end{array}$$

with the **conditional mutual information** *I*[*G* : *A*|*S*] defined as

$$\begin{array}{l} I\left[G:A|S\right]=\langle \log\frac{q\left(a|g,s\right)}{q\left(a|s\right)}\rangle . \end{array}$$

The conditional mutual information gives the amount of information contributed by *G* about *A* in the presence of *S*, and beyond what is contributed by *S* alone. Now, following previous work (van Dijk and Polani, 2013; Genewein et al., 2015), we can define a family of computation costs by taking a weighted sum of the information from the two sources:

(8) $$\begin{array}{l} \text{Computation cost}=\alpha I\left[S:A\right]+\left(1-\alpha \right)I\left[G:A|S\right], \end{array}$$

where α ∈ [0, 1] represents the relative cost of using information from *S* as opposed to information from *G* conditional on *S*. In order to model PWI, it turns out that the minimal information penalty required in the control objective is on the mutual information *I*[*G* : *A*|*S*]—the amount of information that must be “transmitted” from the behavioral goal *G* to specify the action *A* in the context of the perceptual state *S*. So in the computation cost for the PWI simulations, I set α = 0 in Equation (8). The substantive hypothesis here is that there is negligible cost for using information from the perceptual state *S* alone, but high cost for using information from the behavioral goal *G* in the context of the perceptual state *S*.

Defining computation cost in this way, the speaker's production policy is a minimum of the control objective:

(9) $$\begin{array}{l} L\left(q\right)=\langle d\left(a_{s}^{g},a\right)\rangle +\frac{1}{\gamma }I\left[G:A|S\right], \end{array}$$

where $a_{s}^{g}$ indicates the correct action to be taken in state *s* with goal *g*, and *d* : *A* × *A* → ℝ^(+)^ is a semantic distance measure on production actions *A*, as defined in section 3.2. The minima of the control objective in Equation (9) have the form:

(10) $$\begin{array}{l} q\left(a|g,s\right)=\frac{1}{Z\left(g,s\right)}q\left(a|s\right)\exp\left\{-\gamma d\left(a_{s}^{g},a\right)\right\}\\ \;q\left(a|s\right)=\underset{g}{\sum }p\left(g|s\right)q\left(a|g,s\right)\\ \;Z\left(g,s\right)=\underset{a}{\sum }q\left(a|s\right)\exp\left\{-\gamma d\left(a_{s}^{g},a\right)\right\}. \end{array}$$

Below, I will first analyze the policy in Equation (10) and show that it demonstrates semantic interference under reasonable default parameter settings in a simulation of the PWI task, and then that it can capture some of the major qualitative empirical patterns observed in PWI studies when we vary the parameters of the simulation.

### 4.4. Simulation Setup

I model the basic PWI task with the following setup. An agent has access to a behavioral goal and a perceptual state, and produces an output action in response to these. The perceptual state consists of a picture and a written word. The behavioral goal specifies whether the agent should read the word or name the picture. Each word and each picture is associated with a single appropriate target action.

More formally, the behavioral goal is a random variable *G* that can take one of two values, *g* ∈ {name, read}, with the probability of the goal being name equal to a parameter $p_{\text{name}}=\frac{1}{10}$, the same value used in Zénon et al. (2019). This low probability is meant to reflect the fact that when one sees some text, the relevant behavioral goal is usually to read the text, not name the object it is displayed or written on, especially when reading a card or a computer screen in a lab environment. As we will see, this low probability will end up driving the asymmetry between reading and naming in the model.

The perceptual state is represented by the random variable *S* and takes values in *pairs* of discrete objects 〈*w, p*〉, representing a state where an agent is seeing word *w* superimposed on picture *p*. The number of possible words is *N*_*w*_ and the number of possible pictures is *N*_*p*_; in all the simulations below, I fix *N*_*w*_ = *N*_*p*_ = 32 and assume a uniform distribution on the possible states. The output actions are represented by a random variable *A* taking one of *N*_*a*_ = 32 different values. Each goal *g* and state *s* is associated with a target action $a_{s}^{g}$ defined as follows: given the goal *g* = read and the state *s* = 〈*w, p*〉, the target action is *w*; given the goal *g* = name, the target action is *p*. The distance metric among output actions *d* : *A* × *A* → ℝ^(+)^ will be defined below, either as an idealized metric or as a metric derived from word embeddings (Mikolov et al., 2013), when we move to modeling experimental data.

The last parameter we need to specify an RDC policy is the scalar γ, which gives the computational resources (inverse cost) available for information processing in the model. With all these parameters in hand, we can compute the RDC policy from the control objective in Equation (9). Simulation parameters are summarized in Table 1.

**Table 1:** Default parameters of the simulation of the Stroop task.

**Parameter**	**Value**	**Meaning**
*p*_name_	0.1	A priori probability of the behavioral goal being to name, rather than read.
*N*_*w*_	32	Number of different words in possible perceptual states.
*N*_*p*_	32	Number of different pictures in possible perceptual states.
γ	4	Information processing resources (see Equation 9).

*See text for discussion*.

As a more concrete example, suppose the goal *g* = name, and the perceptual state is the pair 〈apple, pear〉, representing the word “apple” superimposed on a picture of a pear. Because the goal is *g* = name, the target action $a_{s}^{g}$ is to say “pear.” If the agent takes this action, then the distortion is zero, because *d*(pear, pear) = 0. On the other hand, if the agent takes the action of saying “apple,” then the distortion is *d*(pear, apple), which may be small, since these are semantically related words that share many features. Because this distortion is low, an agent may be attracted toward saying “apple,” which has higher distortion than “pear,” but has lower computation cost because it does not require attending to the costly behavioral goal. Then the probability of producing the correct word “pear” will be low and the decision cost for the correct word “pear” will be high.

Given a state 〈*w, p*〉 and a goal *g*, we can define one part of the state as the “target” and another as the “distractor.” When *g* = name, the target is *p* and the distractor is *w*. When *g* = read, the target is *w* and the distractor is *p*. In each state, there will be a certain semantic distance between the target and distractor, called the **distractor distance**. If *a*_*w*_ represents the action associated with *w* and *a*_*p*_ is the action associated with *p*, then when *g* = name, the distractor distance is *d*(*a*_*p*_, *a*_*w*_); when *g* = read, the distractor distance is *d*(*a*_*w*_, *a*_*p*_).

The major conditions of PWI experiments are the congruent, semantic, neutral, and unrelated conditions (defined in Figure 2). So far, we have the ability to model three of these: the congruent condition corresponds to the case where the distractor distance is 0 (i.e., the target actions are identical across goals: *a*_*w*_ = *a*_*p*_); the semantic condition corresponds to the case where distractor distance is low; and the unrelated condition means the distractor distance is high. I will return to the neutral condition below.

### 4.5. Results

#### 4.5.1. Basic Results: Idealized Semantic Distance Metric

**Figure 4:**
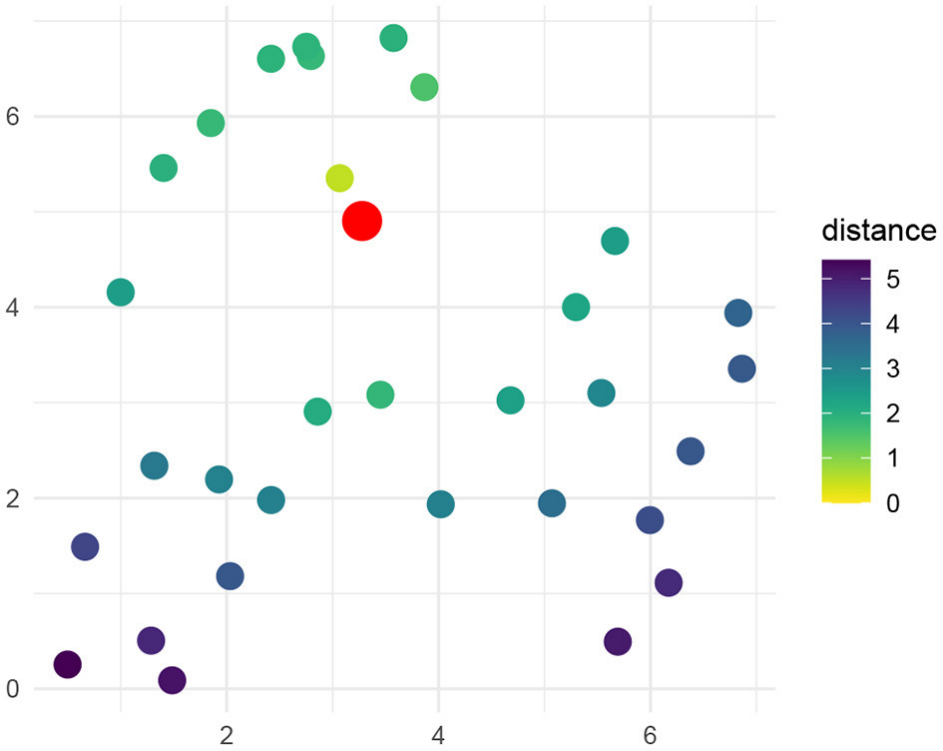
Example of an idealized semantic metric of words as used for basic simulations. Thirty-two words are placed randomly in a two-dimensional bounded Euclidean space of size 7 × 7. A target word is indicated in red. The remaining points are colored according to their distance from the target word.

First I present simulation results showing the existence of semantic interference effects given an idealized semantic metric among words. This metric is generated randomly by placing *N*_*w*_ = 32 words uniformly at random in bounded 2-dimensional space of size 7 × 7. An example such space is shown in Figure 4. An RDC policy was computed for picture naming and word reading given this space, considering all possible pairings of words as pictures and as names.

**Figure 5:**
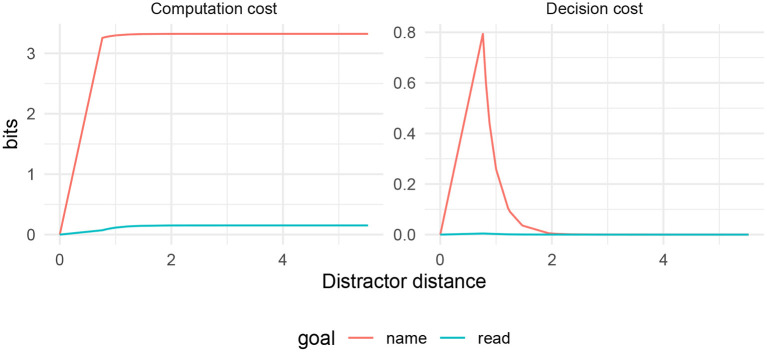
Simulated costs in Picture–Word Interference task, as a function of semantic distance between target and distractor.

In Figure 5, I show the decision cost and the computation cost based on the simulation in this space, as a function of distractor distance. We see a few basic patterns:

- There is no decision cost and low computation cost when the distractor distance *d* = 0, corresponding to the congruent condition in experiments.
- Semantic interference exists in the decision cost. The interference is high for close words (corresponding to the semantic condition), and falls off rapidly at distant words (corresponding to the unrelated condition).
- When the goal is *g* = read, interference of any kind is negligible.

In the simulation, computation cost comes out to be essentially a constant function of the goal, except when the appropriate actions given the two goals coincide (distractor distance 0). In fact, as the distractor distance gets large, the computation cost turns out to approximate the surprisal of the goal given the state −log *p*(*g*|*s*). In doing so, the computation cost recovers the model of Stroop interference from Zénon et al. (2019)^3^.

This most basic simulation already captures several qualitative patterns from the empirical literature (as listed by MacLeod, 1991). First, we recover the fact that naming is generally slower than reading (Cattell, 1886), as indicated by the uniformly higher computation cost for naming. Second, we recover the existence of facilitation in the congruent condition, reflected in lower decision cost and lower computation cost when distractor distance is zero. Third, we recover the existence of interference in the semantic condition relative to the congruent condition and the unrelated condition, as reflected in the decision cost. Fourth, interference exists for the naming task but is negligible in the reading task. Fifth, the interference effect is gradient (Klein, 1964): when the distractor is *more* semantically similar to the target, there is more interference; this is reflected in the decision cost for the naming condition.

The semantic gradient deserves a bit more discussion. There has been controversy in the literature on Picture–Word Interference about whether a semantic gradient really exists, as opposed to a categorical effect for distractors that are in the same category as the target (Hutson and Damian, 2014; Bürki et al., 2020). In the RDC model, there is a semantic gradient observable in the decision cost, but it falls off very rapidly from distance 1 to distance 2, and distance 2 shows only barely more interference than distance 3. Therefore the theory predicts that a semantic gradient does exist, but it is highly concentrated, and might be hard to detect in experiments.

Above, I have shown that RDC can capture the basics of semantic interference in PWI tasks in a simulation with simple and reasonable default parameter settings. Next, I will show how we can recover more of the empirical patterns by varying the parameters of the simulation and the model.

#### 4.5.2. Reverse Stroop

The **reverse Stroop effect** refers to a reversal in the difference between naming and reading in a PWI/Stroop task. Usually, interference happens in the naming task and not in the reading task. However, after a great deal of experience with naming in incongruent trials, two things happen: the interference effect in naming shrinks, and subjects begin to show an interference effect in reading as well as naming (Stroop, 1935; MacLeod, 1991).

While early work hypothesized that the reverse Stroop effect is caused by practice and task familiarity (Stroop, 1935), later work has shown that reverse Stroop effects are more likely related to the difficulty of task switching between naming and reading (Allport and Wylie, 2000; Roelofs, 2021). In terms of simulation parameters, it seems sensible to identify reverse Stroop manipulations with an increase in the parameter *p*_name_, reflecting increased relevance of the naming goal, perhaps due to recency.

**Figure 6:**
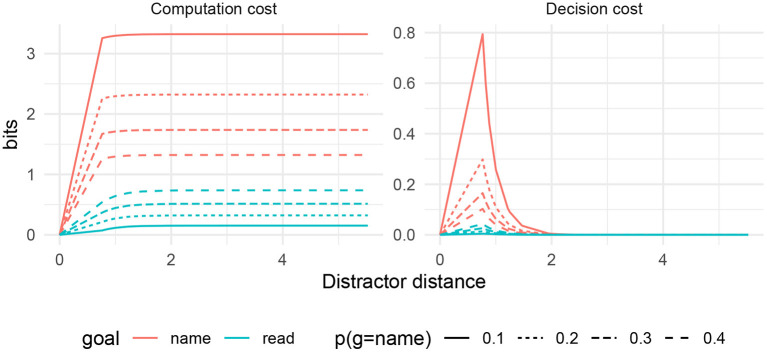
Computation and decision cost for PWI under varying values of *p*_name_. A reverse Stroop effect emerges in the decision cost under the reading goal.

Figure 6 shows computation and decision costs under varying *p*_name_ in the idealized semantic distance metric. As this value increases, a reverse Stroop effect emerges: the reading task begins to show interference in both costs. Meanwhile, the interference associated with naming is predicted to decrease.

Beyond the Reverse Stroop effect, the simulations here demonstrate the general effects of varying the simulation parameter *p*_name_. Such results could be used, for example, when modeling picture–picture interference effects, where participants are confronted with two pictures and must name only a certain one (for example, Glaser and Glaser, 1989). In that case, the behavioral goals associated with each of the two pictures would have more similar prior probabilities, and the resulting RDC predictions would look more like the dotted lines in Figure 6.

#### 4.5.3. Empirically-Derived Semantic Distance Metric

The results above showed basic qualitative effects in an idealized semantic space. Now I turn to results based on an empirically-derived semantic space, leading to a quantitative comparison to human reaction times. The use of an empirically-derived semantic space brings two advantages over the idealized space above: (1) it allows for a comparison with experimental data on real words, and (2) it shows that the predicted interference effects arise given a realistic geometry for the semantic space and a realistic distribution of words in it.

In the last decade, the field of natural language processing has devoted a great deal of attention to deriving representations of words as points (called **embeddings**) in high-dimensional space, such that the distances among embeddings reflect semantic relationships among words (Mikolov et al., 2013; Pennington et al., 2014). These representations differ in their details, but they are all derived by an optimization process whose goal is to create embeddings such that the *context* of a word can be predicted accurately from its embedding (Goldberg and Levy, 2014), in keeping with the old linguistic intuition that the meaning of a word is related to its distribution with respect to other words (Harris, 1954; Firth, 1957). The result is that the “distance” between two words *A* and *B* reflects the difference between the typical contexts for *A* and *B*. As such, these distributional embeddings provide a distance metric which fits with the RDC framework, which holds that two actions are similar if there is low cost for failing to distinguish them. In particular, the embedding distance between words reflects how badly one would mis-predict the context of one word when it is mistaken for another.

There have been previous attempts to model semantic interference effects in Stroop and PWI using embedding spaces, such as these (de Marchis et al., 2013; Hutson and Damian, 2014). The embedding spaces can broadly distinguish between semantically close words compared against unrelated words, although they do not seem to be able to make reliable item-level predictions within semantically close words (Hutson and Damian, 2014).

Here, I adopt the English fastText embedding space derived by Facebook^4^ as a semantic distance metric among words. In work using these embeddings, the distance between embeddings *u* and *v* is usually quantified as **cosine distance**:

$$\begin{array}{l} d_{\text{cos}}\left(u,v\right)=1-\frac{u\cdot v}{{\left\|u\right\|}_{2}{\left\|v\right\|}_{2}}, \end{array}$$

where · indicates a dot product and ||*u*||_2_ indicates an *L*_2_ norm. In order to produce distances in the interval [0, ∞), I apply a logit transform to the cosine distance^5^.

I use the set of 32 words from the Picture–Word Interference experiment presented in Roelofs and Piai (2017). The items from this experiment consist of picture–word pairings which are either semantically close (“semantic”) or semantically unrelated (“unrelated”). Here, I show that RDC with the fastText embedding space predicts higher cognitive cost for the semantic pairings as opposed to the unrelated word pairings, and also lower cost when the word and the picture to be named are identical^6^. Except for the semantic distance metric, all other parameters of the simulation are the same as above.

**Figure 7:**
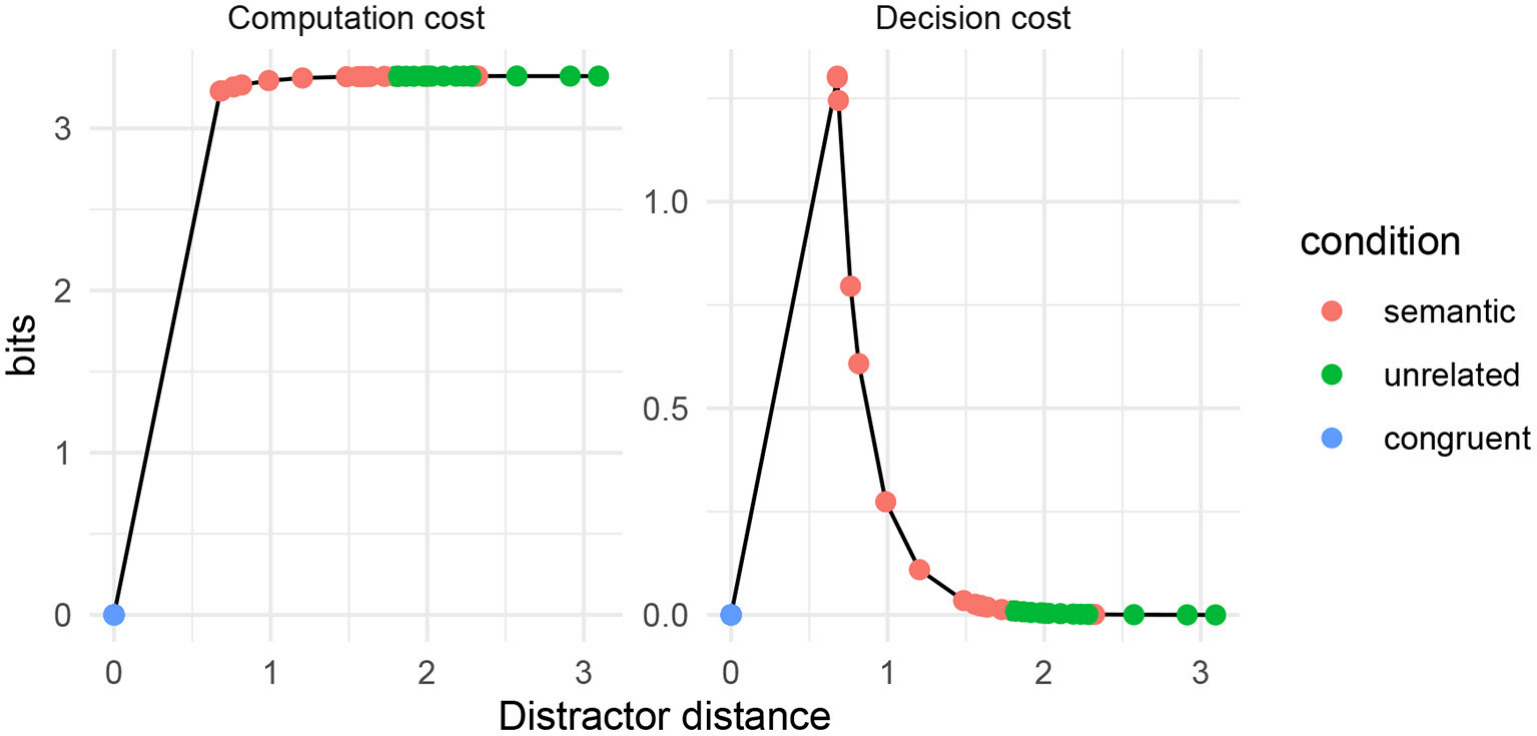
Computation and decision costs for word pairs from the items of Roelofs and Piai (2017), using fastText as the semantic distance metric.

In Figure 7, I show theoretical computation cost and decision cost by distractor distance for the word pairs listed in Roelofs and Piai (2017). Red dots indicate word pairs in the “semantic” condition; green dots indicate word pairs in the “unrelated” condition; and blue dots indicate identical words. Predicted cognitive cost is lowest for identical words. For “unrelated” and “semantic” words, there is high computation cost. For “semantic” words, there is also high decision cost.

The simulation using an empirically-derived semantic distance metric shows the same qualitative patterns as the simulation using an idealized metric. Furthermore, we see that the semantic distances largely correspond (although imperfectly) with the designation of items as “semantic” vs. “unrelated.”

#### 4.5.4. Neutral vs. Unrelated Trials

The PWI task has a fourth major condition: the *neutral* condition, where a picture is presented along some kind of neutral orthographic stimulus that would not reasonably be read out loud, such as XXXXX. Here, I will incorporate this condition into the simulation and show that we immediately recover three empirically-attested patterns: (1) there is facilitation in the congruent condition relative to the neutral condition, (2) there is interference in the unrelated condition relative to the neutral condition, and (3) the size of facilitation is small relative to the size of interference (MacLeod, 1991).

Recall that in the basic simulation, the a priori probability that the behavioral goal is *g* = name rather than *g* = read is $\frac{1}{10}$. I model the neutral condition by adding into the simulation a set of states *s*_neutral_ with neutral text distractors, such that $p\left(g=\text{name}|s_{\text{neutral}}\right)=\frac{9}{10}$ for all neutral states. This models the scenario where a subject sees XXXXX superimposed on an image. The idea is that given such a state, a subject would only expect to actually read the stimulus (saying “eks eks eks eks eks”) $\frac{1}{10}$ of the time. Outside of a state with a neutral distractor *s*_neutral_, the probability of naming is still $\frac{1}{10}$.

**Figure 8:**
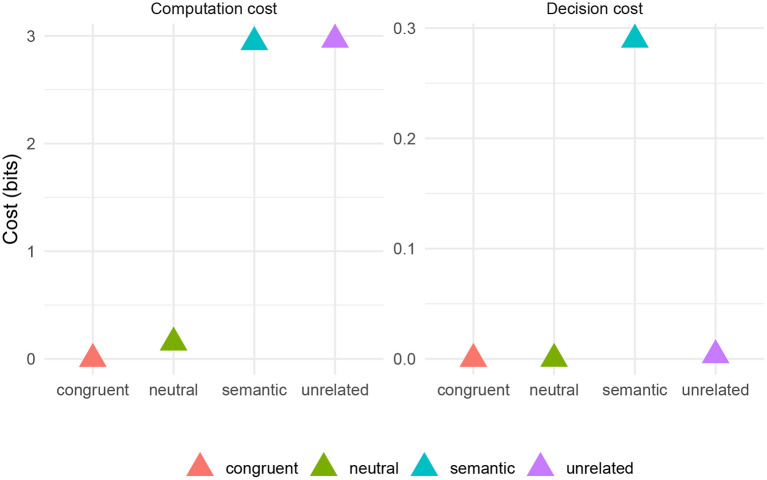
Simulated costs by PWI task condition based on materials from Roelofs and Piai (2017) and fastText word embeddings.

Figure 8 shows the simulated decision and computation costs for four experimental conditions based on the items from Roelofs and Piai (2017): congruent (the case where the distance *d* = 0), semantic, unrelated, and neutral (simulated as the case where *s* = *s*_neutral_). The three empirical patterns are captured here by the computation cost. The neutral condition has drastically reduced computation cost relative to the semantic and unrelated conditions, indicating facilitation. Also, the computation cost is slightly less in the congruent case relative to the neutral case, indicating facilitation. Also, the size of the facilitation effect (the difference between neutral and congruent conditions) is small relative to the interference effect (the difference between neutral and semantic/unrelated conditions).

The model robustly recovers the existence of facilitation and interference. The relative magnitude of facilitation and interference depends on a model parameter: the probability *p*(*g* = name|*s* = *s*_neutral_)^7^. Therefore, it is therefore possible to make a prediction: the facilitation effect should get larger under any manipulation that makes the orthographic string in the neutral condition more and more like something that someone would reasonably read. In fact, there is already some evidence in this direction in the literature: pseudowords, which presumably fall somewhere between XXXXX and a real word in terms of *p*(*g* = name|*s*), cause less interference than real words in the Stroop task (Klein, 1964).

#### 4.5.5. Fit to Human RT Data

Here I relate the simulated computation and decision costs to empirical human RT data. To do so, we need a more specific linking function from computation and decision cost to RT.

I propose that RT can be predicted from a linear combination of computation and decision cost. That is, the predicted RT in a condition is given from cognitive costs by a transformation:

$$\begin{array}{l} \text{RT}=a+bX+cY, \end{array}$$

where *X* is computation cost, *Y* is decision cost, and *a*, *b*, and *c* are non-negative scalars. This linking function supposes that computation cost and decision cost are each associated with some fixed rate of information processing, given by *b* and *c*, respectively, in terms of milliseconds per bit. The scalar *a* represents a constant RT delay across conditions (in the model of Zénon et al., 2019, this constant cost corresponds to perceptual information processing).

**Figure 9:**
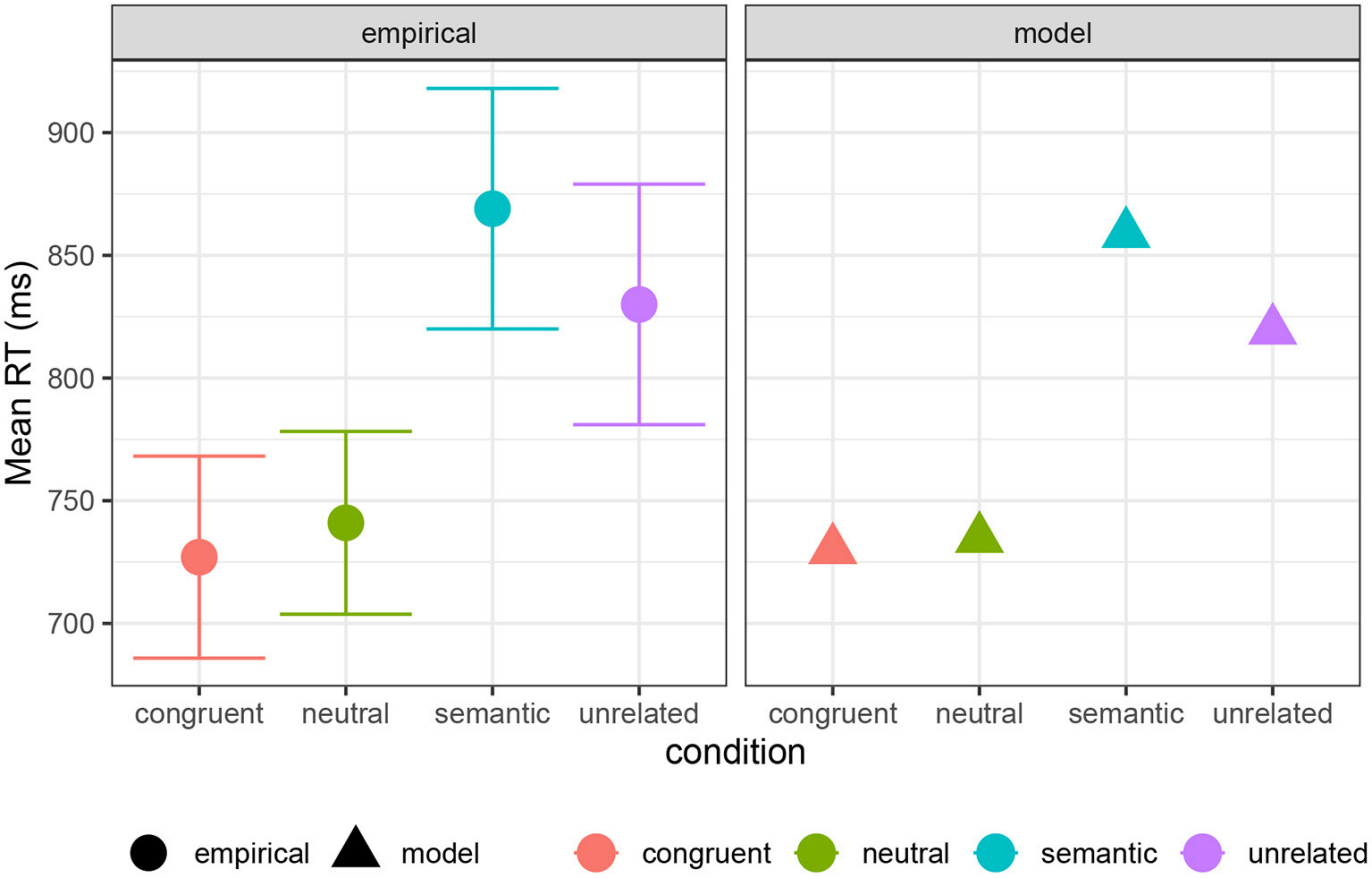
Empirical mean RTs for PWI conditions from Roelofs and Piai (2017), compared with model predictions (see text). Error bars show 95% confidence intervals of the mean in the empirical data.

Figure 9 shows a comparison of empirical mean RTs in a PWI task, drawn from Roelofs and Piai (2017), compared against simulated RTs, with *a* = 730 ms, *b* = 30 ms/bit, and *c* = 140 ms/bit^8^. This mixture gives a good qualitative fit to the human data.

The relationship of information-processing costs to RT may not be so simple, however. In particular, RT distributions appear to follow what is called an Ex-Gaussian distribution (Ratcliff, 1979; Luce, 1986; Balota et al., 2008). An Ex-Gaussian random variable is the sum of a Gaussian random variable with mean μ and an Exponential random variable with rate τ. The resulting distribution is skewed positive when compared with a Gaussian distribution. Interestingly, it has been suggested that the μ and τ parameters of the Ex-Gaussian distribution reflect different aspects of cognitive processing in the PWI task (Heathcote et al., 1991; Mewhort et al., 1992; Spieler et al., 2000; Piai et al., 2011, 2012; Roelofs, 2012; Scaltritti et al., 2015; San José et al., 2021).

**Figure 10:**
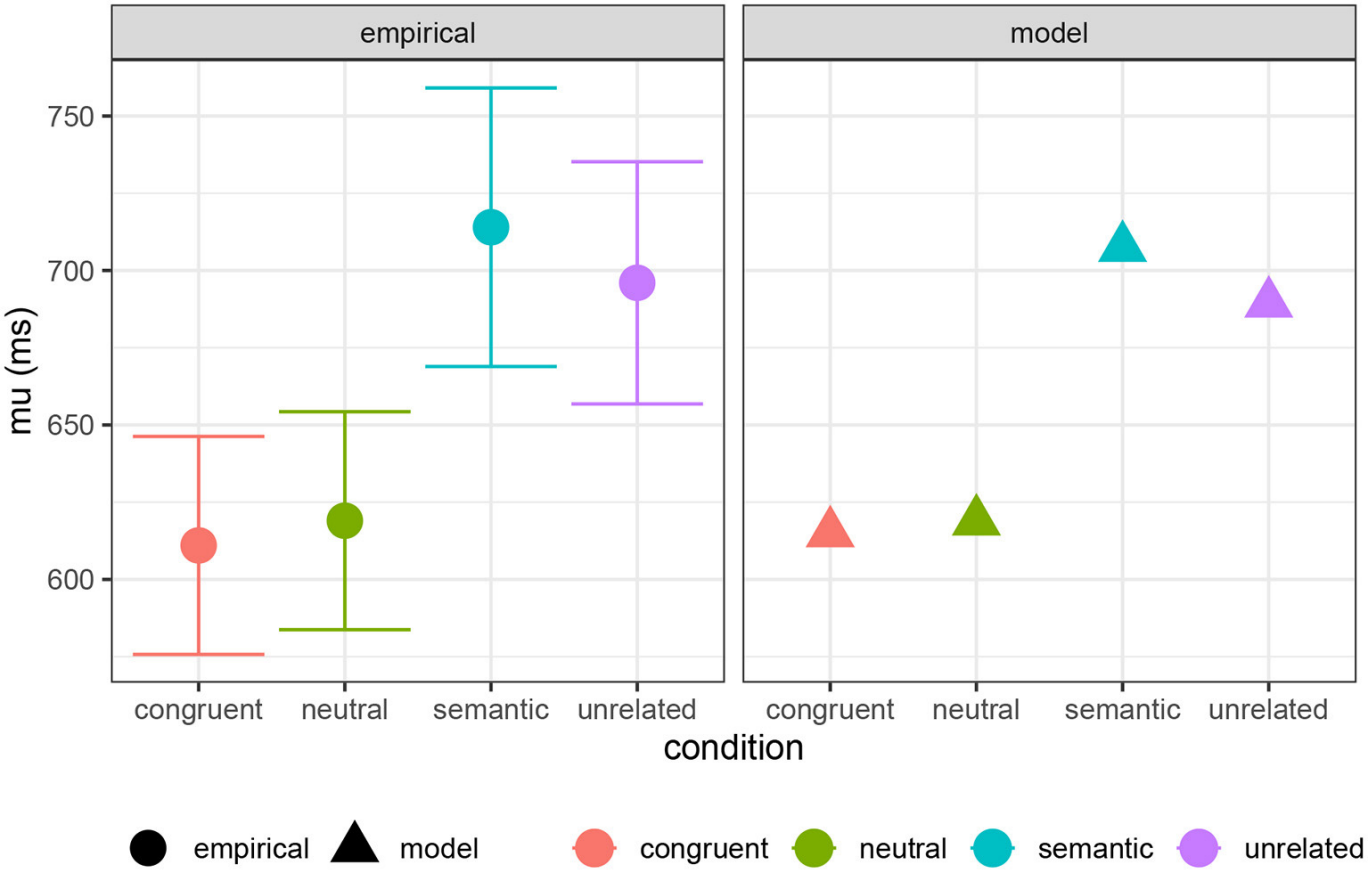
Empirically estimated μ parameter of Ex-Gaussian RT distribution for PWI conditions from Roelofs and Piai (2017), compared with model predictions (see text).

**Figure 11:**
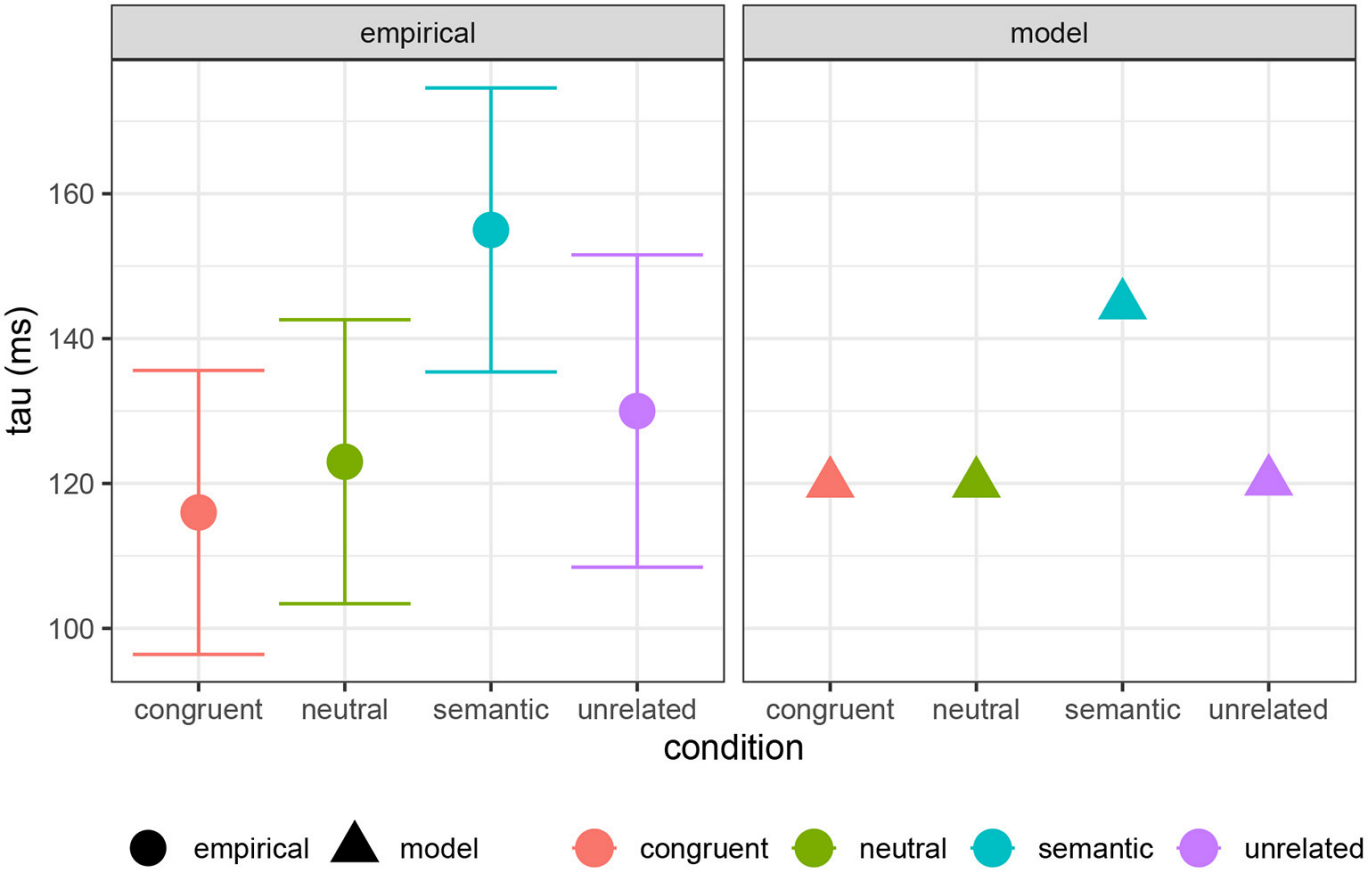
Empirically estimated τ parameter of Ex-Gaussian RT distribution for PWI conditions from Roelofs and Piai (2017), compared with model predictions (τ = 120 + 85 × Decision cost).

Here I present an analysis comparing computation and decision costs to the full Ex-Gaussian analysis of experimental PWI data, including congruent, semantic, neutral, and unrelated conditions, performed by Roelofs and Piai (2017). In Figure 10, I show their estimates of the μ parameter compared with a combination of computation cost and decision cost (*a* = 615 ms, *b* = 25 ms/bit, *c* = 65 ms/bit). In Figure 11, I compare their τ estimates to decision cost alone (*a* = 120 ms, *b* = 0, *c* = 85 ms/bit)^9^. The reasonable qualitative match suggests that both computation and decision cost are reflected in the μ component of the RT distribution, while only decision cost is reflected in the τ component. It is striking that the τ component seems to reflect only decision cost, suggesting that decision cost is indeed an index of a distinct kind of cognitive cost. This result is in line with the pattern reported by Roelofs and Piai (2017): μ shows a contrast among neutral, unrelated, and semantic conditions, while τ shows a contrast only between the semantic condition and the others (see also Scaltritti et al., 2015; San José et al., 2021).

Summing up, the overall empirical pattern is that computation cost captures basic interference effects in RT, while decision cost captures the additional RT slowdown associated with semantically close distractors. The RT component μ reflects both computation and decision cost, while the additional RT component τ reflects only decision cost.

### 4.6. Discussion

It is striking that the framework laid out here can successfully model many aspects of PWI, despite being developed nearly entirely for purposes other than cognitive modeling. Rate–distortion theory was developed purely as an abstract theory of lossy communication, and its application to control problems has primarily been confined to the computer science and robotics literature.

Furthermore, RDC captures the major empirical patterns of the Picture–Word Interference task with few free parameters. The degrees of freedom in the specification of the model are (1) the distribution over goals and states, (2) the information-processing resource parameters used to define the control objective (the scalar γ, which was set to a constant value in all simulations reported above), and (3) the similarity metric among actions. All of these degrees of freedom correspond to quantities that can be independently estimated, at least in principle. The distribution over goals and states is set by the frequency of goals and states in a person's everyday experience; the information-processing cost parameters are set by studies of cognitive difficulty; and the similarity metric among actions is determined by the relative cost of the consequences of confusing one action for another. The result is a parsimonious model that captures several patterns naturally.

## 5. General Discussion

I have shown that the rate–distortion theory of control can naturally account for similarity-based interference in general, and that it offers a strong model of Picture–Word/Stroop interference effects. Now I turn to the interpretation of the model and how it relates to word production more generally.

### 5.1. Interpretation of Computation and Decision Cost

I used two notions of cost: computation cost and decision cost, where computation cost is the cost term that is contained in the control objective, and decision cost is the surprisal of selecting a single action given a probabilistic policy. As a summary, semantic similarity-based interference emerged in the decision cost, while computation cost predicted general interference and difficulty for the less-probable goal in context (naming as opposed to reading).

I proposed that computation cost and decision cost map linearly to RT. The reason for this proposal was simplicity. However, it may be that other linking functions provide a better connection between *q*(*a*|*g, s*) and empirically observable response times, for example by linking RDC components to components of drift–diffusion models (Bogacz et al., 2006; Ortega and Braun, 2013). I leave the exploration of this possibility to future work.

### 5.2. Relation to Algorithmic-Level Models

As a computational-level theory, RDC specifies only the problem being solved by our cognitive system, and does not make claims about algorithmic or implementational details. It should be hoped, then, that existing successful algorithmic models of PWI can be seen as implementing the core parts of the RDC account.

In this connection, the recent extension of WEAVER++ by San José et al. (2021) is especially interesting, as it adds an element of periodically lapsing attention to the behavioral goal in order to explain the Ex-Gaussian distribution of RTs in PWI experiments. Similarly, the RDC model of picture–word interference crucially works by positing a cost associated with extracting information from the behavioral goal in the presence of the perceptual state. Essentially, the RDC agent can only access the behavioral goal through a channel with limited bandwidth. This limited bandwidth equates to a kind of inattention: because the agent has limited resources with which to attend to the channel, it will often not attend. Indeed, RDC was initially introduced as a model of “rational inattention” in economics with this reasoning (Sims, 2003, 2005, 2010).

Similarly, the production rules and spreading activation dynamics of WEAVER++ can be seen as implementing RDC-like behavior. For example, one production rule used in the WEAVER++ simulation of PWI in San José et al. (2021) states that if the behavioral goal is to name a picture, and a written word is present, then activation relating to the written word is blocked off. Similar logic is instantiated by the RDC policy. Consider the equilibrium probability (following Equation 10) to produce the written word *a*_*w*_ when the behavioral goal is *g* = name:

$$\begin{array}{l} q\left(a_{w}|g=\text{name},s\right)\propto q\left(a_{w}|s\right)\exp\left\{-\gamma d\left(a_{p},a_{w}\right)\right\}, \end{array}$$

where *a*_*p*_ is the action corresponding to naming the picture. The first factor *q*(*a*_*w*_|*s*) will be relatively large, because the prior is that the behavioral goal is usually to read, not to name. This large value corresponds to activation for the written word. However, this large value will be squashed by the exponentially small value of the second factor exp{−γ*d*(*a*_*p*_, *a*_*w*_)} (unless *a*_*p*_ and *a*_*w*_ are close), resulting in an ultimately low probability to name the written word. This corresponds to blocking of activation.

The RDC model presented here shows how similarity-based interference can arise from a very generically-defined computational bottleneck. It achieves this generality without sacrificing quantitative precision. Nevertheless, it is likely that many aspects of PWI and similarity-based interference more generally might only be explainable within more algorithmic and mechanistic frameworks. For example, a great deal of work on PWI has dealt with stimulus-onset asynchrony (SOA) effects, where the distractor word or the picture do not appear at the same time. These effects are naturally captured in spreading-activation models that describe the evolution of activation with time. It is less clear how such time-based effects would be captured within a purely computational-level account, which simply models the *function* that is computed by cognitive systems, and not *how* it is computed.

### 5.3. Further Word Production Phenomena: Facilitation

I intend to advance RDC, or an extension of it, as a model of word production in general. I have presented its application to interference in PWI and Stroop paradigms because these are well-known and challenging phenomena to model. However, there are many other language production phenomena on which an RDC model has yet to be tested, including several that arise within the PWI paradigm. One such set of phenomena is facilitation, both phonological and semantic.

The PWI task exhibits phonological facilitation, meaning that naming time is sped up when the distractor word is *phonologically* similar to the target word (Meyer and Schriefers, 1991). In the simple simulations presented here, the RDC does not predict this kind of facilitation. However, it can when the control objective is specified in more detail, as I sketch below.

**Figure 12:**
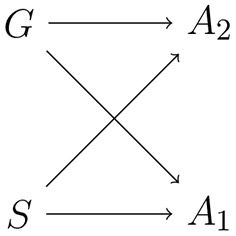
Schematic of a policy where the behavioral *G* and the perceptual state *S* determine two actions *A*_1_ and *A*_2_ to be performed by different actuators.

Imagine that the goal of the policy is not to output a single atomic output action, but rather to output a large number of actions. For example, one can imagine that the policy must output instructions to a large number of actuators. This kind of policy is illustrated in Figure 12. Equivalently, the output of the policy is a vector **a** = [*a*_1_, *a*_2_, …, *a*_*n*_] of actions to be performed by *n* different actuators.

Given this kind of policy, we can define a “phonological” similarity metric among actions **a**_1_ and **a**_2_ in terms of how many elements overlap between **a**_1_ and **a**_2_. For each overlapping element, we will have a facilitation effect, and for each non-overlapping element, we will have an interference effect. The result is overall facilitation when the target action and the distractor have more overlapping elements.

There are other extensions of RDC and other mechanisms that could give rise to facilitation effects, for example multi-stage hierarchical policies where the output of one policy becomes the input to another. Such families of more elaborate RDC policies have been explored in simulations by Genewein et al. (2015).

Facilitation has also been reported in PWI settings for certain semantically similar words, and a great deal of effort has gone into experimentally characterizing when semantically similar words will cause facilitation or interference, often dealing with whether a given target word is in the “response set” for the experiment (e.g., Roelofs, 1992, 2003; Caramazza and Costa, 2000, 2001; Mahon et al., 2007; Piai et al., 2012). While empirical picture remains complex (Bürki et al., 2020), these results have often been taken to reflect dynamics during different stages of word production. While the simple RDC model presented here does not predict these facilitation effects, a more articulated model might: for example, a model with a non-zero penalty on perceptual state information, or a hierarchical policy (Genewein et al., 2015; Zénon et al., 2019). The answer may also lie in the linking function from the RDC policy to observables, such as RT: if computation cost is sometimes the dominant determinant of reaction times, rather than decision cost, then Figure 5 suggests that we would expect semantic facilitation rather than interference. I leave the investigation of these possibilities to future work.

### 5.4. Conclusion

This work has extended the reach of information-theoretic models of language processing. Although information-theoretic models have seen broad success in the study of language comprehension (Hale, 2001; Moscoso del Prado Martín et al., 2004; Levy, 2008; Hale et al., 2018; Futrell et al., 2020) and the emergence of linguistic structure (Zaslavsky et al., 2018; Hahn et al., 2020), they have not yet seen much application to language production. This work has taken the first steps toward remedying this gap using the rate–distortion theory of control.

Furthermore, the apparent inability to capture similarity relations among stimuli has been a major barrier for the adoption of information-theoretic models in cognitive science (Luce, 2003, p. 185). This work shows that rate–distortion theory allows us to overcome this difficulty and model some of the most salient similarity-based effects in psychology.

## Open Practices Statement

All data and code for reproducing the results in this paper can be found online at http://github.com/langprocgroup/wordprodmodel.

## Data Availability Statement

The original contributions presented in the study are included in the article/supplementary material, further inquiries can be directed to the corresponding author/s.

## Author Contributions

RF conceived the research, conducted the research, and wrote the paper.

## Conflict of Interest

The author declares that the research was conducted in the absence of any commercial or financial relationships that could be construed as a potential conflict of interest.

## Footnotes

^1^The function *d*(*a*_*s*_, *a*_*d*_) is technically a pre-metric. It satisfies *d*(*a, a*) = 0 for all actions *a*, and it is always non-negative. It is non-negative because *a*_*s*_ is defined as the action with minimal cost in state *s*. The function is only a pre-metric, not a full metric, because it is not generally symmetrical. That is, *d*(*a*_*s*_, *a*_*d*_) ≠ *d*(*a*_*d*_, *a*_*s*_) in general.

^2^The probability of the target action *q*(*a*_*s*_|*s*) is calculated as follows:

$$\begin{array}{l} q\left(a_{s}|s\right)=\frac{q\left(a_{s}\right)\exp\left\{-\gamma d\left(a_{s},a_{s}\right)\right\}}{q\left(a\right)\exp\left\{-\gamma d\left(a_{s},a_{d}\right)\right\}+q\left(a_{d}\right)\exp\left\{-\gamma d\left(a_{s},a_{d}\right)\right\}}\\ \;=\frac{q\left(a_{s}\right)\exp\left\{0\right\}}{q\left(a_{s}\right)\exp\left\{0\right\}+q\left(a_{d}\right)\exp\left\{-\gamma d\left(a_{s},a_{d}\right)\right\}}\\ \;=\frac{q\left(a_{s}\right)}{q\left(a_{s}\right)+q\left(a_{d}\right)\exp\left\{-\gamma d\left(a_{s},a_{d}\right)\right\}}\\ \;=\frac{1}{1+\frac{q\left(a_{d}\right)}{q\left(a_{s}\right)}\exp\left\{-\gamma d\left(a_{s},a_{d}\right)\right\}}. \end{array}$$

This is an instance of the general logistic curve

$$\begin{array}{l} f\left(x\right)=\frac{1}{1+\exp\left\{-k\left(x-x_{0}\right)\right\}} \end{array}$$

with slope parameter *k* = γ and initial condition $x_{0}=\frac{1}{\gamma }\log\frac{q\left(a_{d}\right)}{q\left(a_{s}\right)}.$ More generally, given a set of distractors *a*_*d*_ ≠ *a*_*s*_, the probability of the correct action *a*_*s*_ is

$$\begin{array}{l} q\left(a_{s}|s\right)=\frac{1}{1+\sum_{a_{d}\neq a_{s}}\frac{q\left(a_{d}\right)}{q\left(a_{s}\right)}\exp\left\{-\gamma d\left(a_{d},a_{s}\right)\right\}}. \end{array}$$

^3^When distractor distance is 0, computation cost comes out to nearly zero. This may seems surprising, but follows from the fact that computation cost here is the pointwise conditional pointwise mutual information $\log\frac{q\left(a|g,s\right)}{q\left(a|s\right)}$, which is zero when the action *a* is already fully specified by the perceptual state *s*, such that the behavioral goal *g* adds no new information. It should be noted that computation cost zero does not imply a prediction of RT zero—see section 4.5.5.

^4^Available for download at https://fasttext.cc/docs/en/pretrained-vectors.html

^5^The logit-transformed distance metric between two word embeddings *u* and *v* is

$$\begin{array}{l} d\left(u,v\right)=\text{logit}\left(\frac{1}{2}+\frac{1}{2}d_{\text{cos}}\left(u,v\right)\right), \end{array}$$

with the logit function defined as

$$\begin{array}{l} \text{logit}\left(x\right)=\log\left(\frac{x}{1-x}\right). \end{array}$$

^6^These words were originally in Dutch; I translate them into English in order to get their distances. In preliminary experiments, I also tried using the Dutch fastText vectors, and using the English GloVe vectors (Pennington et al., 2014). I use the English fastText vectors because I found that they most reliably assign lower distances to the “semantic” word pairings compared to the “unrelated” word pairings in the experimental items. Rank-order correlations of semantic distances among the embedding spaces are: English fastText vs. English GloVE ρ = 0.77; English fastText vs. Dutch fastText ρ = 0.59; English GloVE vs. Dutch fastText ρ = 0.54.

^7^The default values for *p*(*g*|*s*) have not been tuned to fit the human data, but were selected a priori and kept constant throughout all simulations.

^8^All of the scaling factors presented in this section were derived by linear regression on the empirical means, followed by rounding. From the linear regressions, the optimal models before rounding are

$$\begin{array}{l} \text{mean RT}\approx 737+28\times \text{Computation cost}+139\times \text{Decision cost}\\ \;\mu \approx 615+25\times \text{Computation cost}+65\times \text{Decision cost}\\ \;\tau \approx 123+2\times \text{Computation cost}+87\times \text{Decision cost}. \end{array}$$

^9^The decision to map computation cost to μ and τ, and decision cost to τ alone, was taken *post-hoc* based on regressions on the empirical RTs.
